# Intestinal Motility Dysfunction in Goto-Kakizaki Rats: Role of the Myenteric Plexus

**DOI:** 10.3390/cells13191626

**Published:** 2024-09-28

**Authors:** Gabriela Mandú Gimenes, Joice Naiara Bertaglia Pereira, Eliane Borges da Silva, Alef Aragão Carneiro dos Santos, Thais Martins Rodrigues, Giovanna de Oliveira Santana, Maria Vitoria Martins Scervino, Tania Cristina Pithon-Curi, Sandro Massao Hirabara, Renata Gorjão, Rui Curi

**Affiliations:** 1 Interdisciplinary Post-Graduate Program in Health Science, Institute of Physical Activity and Sports Sciences, Cruzeiro do Sul University, Rua Galvão Bueno, 868, Liberdade, São Paulo 01506-000, Brazil; 2 Butantan Institute, São Paulo 05585-000, Brazil

**Keywords:** constipation, cholinergic neuron, enteric nervous system, Goto-Kakizaki rats, type 2 diabetes mellitus

## Abstract

Diabetes mellitus is associated with changes in intestinal morphology and the enteric nervous system. We previously reported constipation in Goto-Kakizaki (GK) rats, a non-obese model for type 2 diabetes mellitus. Aim: The morpho-quantitative analysis of myenteric plexus neurons in the small and large intestines of 120-day-old male GK rats was investigated. Methods: The diabetes was confirmed by high fasting blood glucose levels. The myenteric plexus was evaluated through wholemount immunofluorescence. The morpho-quantitative analyses included evaluating neuronal density (neurons per ganglion) of the total neuronal population, the cholinergic and nitrergic subpopulations, and enteric glial cells per ganglion. The cell body area of 100 neurons per segment per animal was measured. Results: The total neurons and nitrergic subpopulation were unaltered in the GK rats’ small and large intestines. The cholinergic subpopulation exhibited decreased density in the three segments of the small intestine and an increased number in the proximal colon of the GK rats. The number of enteric glial cells increased in the ileum of the GK rats, which could indicate enteric gliosis caused by the intestinal inflammatory state. The area of the cell body was increased in the total neuronal population of the jejunum and ileum of the GK rats. Frequency histograms of the cell body area distribution revealed the contribution of cholinergic neurons to larger areas in the jejunum and nitrergic neurons in the ileum. Conclusion: The constipation previously reported in GK rats might be explained by the decrease in the density of cholinergic neurons in the small intestine of this animal model.

## 1. Introduction

Impaired insulin action and secretion, glucose intolerance, and hyperglycemia are the major features of type 2 diabetes mellitus (T2DM), a metabolic disorder that accounts for 90% of diabetes cases [1,2,3]. Epidemiological data show that 541 million adults have impaired glucose tolerance, which increases the risk of developing T2DM, and 537 million people around the world have DM [3].

Adipose tissue meta-inflammation promotes a pro-inflammatory state in obese individuals, with an accumulation of immune cells, immunometabolic changes, and the production and secretion of hormones, adipokines, and cytokines, which can lead to the establishment of insulin resistance (IR) and T2DM [4,5,6]. However, despite the classic well-known association between obesity and T2DM, a considerable proportion of diabetic patients are not obese.

Researchers at Tohoku University in Japan developed a spontaneous non-obese T2DM experimental model, the Goto-Kakizaki (GK) rat. This strain was created by the selective breeding of non-diabetic Wistar rats with mild glucose intolerance in the oral glucose tolerance test (GTT) to form a parental group [7,8]. Thus, the GK rats facilitate studying the effects of diabetes per se and the genetic, inflammatory, metabolic, and intestinal microbiota mechanisms involved in the development of T2DM without the complicating factors caused by obesity.

This experimental model of T2DM displays insulin resistance, basal hyperinsulinemia, reduced glucose tolerance, increased plasma cholesterol levels, and increased inflammatory cytokine expression in the liver [9]. Other organs and tissues are also affected by diabetes in GK rats; the function of brown adipose tissue is impaired in GK rats, with evidence of whitening [10]. Additionally, in the brain, glucose uptake is attenuated and accompanied by neuroinflammation [11]. This model also has alteration in the intestine; Gimenes et al. reviewed the morpho-physiology, gut microbiota, and hormone features of the gastrointestinal tract (GIT) of GK rats published so far [12].

Our group studied the small intestine of GK rats, and the results show an increase in the expression of pro-inflammatory cytokines (e.g., IL-1β) and morphological remodeling. A pan-neuronal analysis of the myenteric plexus showed that despite no neuronal loss, there was an increase in the cell body area of myenteric neurons accompanied by delayed intestinal transit compared to the Wistar control rats [13]. These data show that the changes in the myenteric plexus of GK rats need to be further investigated, especially concerning neuronal subpopulations, which are differently affected by diabetes [13].

The impaired intestinal motility in GK rats seems to contribute to the hyperglycemia seen in this experimental model. Ouyang et al. submitted GK rats to intestinal electrical stimulation, and the acute treatment improved postprandial blood glucose, early-phase insulin secretion, intestinal motility, and GLP-1 secretion. The authors argue that postprandial blood glucose improvement is associated with the accelerative effect in the intestinal transit of GK rats [14].

The complications caused by diabetes mellitus include changes in the GIT, which affect 75% of diabetic patients [15,16], with diarrhea, constipation, fecal incontinence, gastroesophageal reflux, gastroparesis, and nausea being the most recurrent symptoms of diabetic patients [17,18]. The GIT is governed by a complex network of neurons and enteric glial cells (EGCs), which form the enteric nervous system (ENS), commonly referred to as the “second brain” and composed of submucosal and myenteric plexuses [19].

Strategically localized between the circular and longitudinal muscle layers, the myenteric plexus manages the reflex circuit of peristaltic movements [20]. Intestinal motility is one of the functions coordinated by the ENS. It requires integrating all its components, the distinct types of neurons and EGCs, and the participation of smooth muscles, epithelium, and interstitial cells of Cajal [20,21,22,23].

Choline acetyltransferase (ChAT) and neuronal nitric oxide synthase (nNOS) stand out as the most abundant neurons and, therefore, the most utilized ENS markers [24]. The EGCs are involved in neuroprotection, motility, and barrier function, in addition to communication with neurons, microbiota, and the immune system [25], and can be studied using calcium-binding protein B (S100B) as a marker [26].

A previous study reported that diabetes is related to damage in the neurons and EGCs in the ENS and that impairments in gastrointestinal function lead to complications and symptoms that worsen the disease [27]. Changes in myenteric neurons of different experimental models of DM (streptozotocin- or diet-induced) were also detected [28,29]. Furthermore, researchers have reported that duodenal hypercontractility leads to IR through a message to the hypothalamus [24,30].

The importance of the intestine in glycemic control, the gastrointestinal symptoms associated with DM, and the reduction in intestinal transit suggest that the intestine of GK rats may be involved in IR and T2DM. Therefore, the present study evaluated the neurons that control intestinal motility. We assessed the neuronal density of the total population of neurons and subpopulations, inhibitory (nNOS+) and excitatory (ChAT+), and the enteric glia (S100+) in the small and large intestines of 120-day-old GK rats, a well-established diabetic model.

## 2. Materials and Methods

### 2.1. Animals

**Figure 1:**
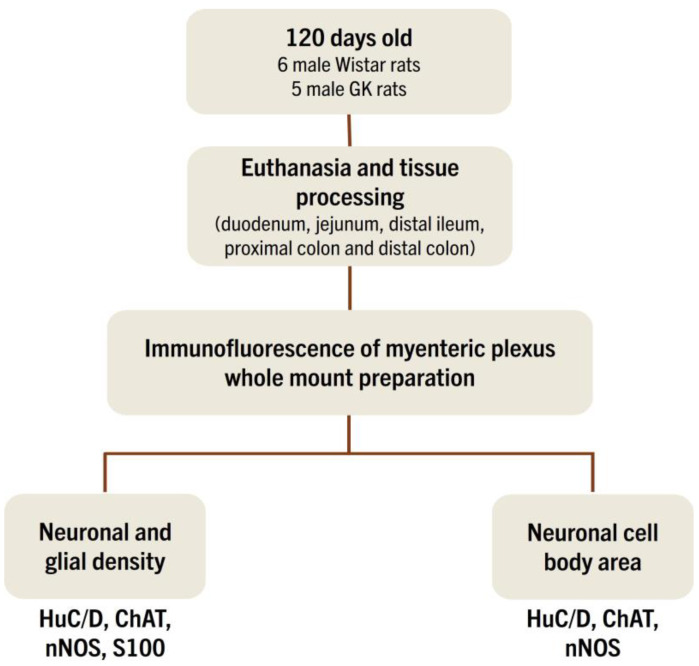
Schematic representation of the animal groups (Wistar and GK), experimental protocol, and analyses. Eleven male rats were randomly allocated into six control (Wistar) and five experimental (GK) groups. At seventeen weeks of age, all 11 control and experimental animals were weighed, euthanized by CO_2_ inhalation, and decapitated. Following euthanasia, the duodenum, jejunum, distal ileum, and proximal and distal colon were collected and processed for morphometrics analysis of the myenteric plexus.

We obtained the Wistar and GK rats from Charles River Laboratories International Inc. (Wilmington, MA, USA). All animals were housed in the Interdisciplinary Post-graduate Program in Health Sciences animal facility at Cruzeiro do Sul University. The rats were kept at 23 ± 2 °C, with a light/dark cycle of 12 h. Both groups of rats had free access to standard rodent chow (Nuvilab ^®^, Curitiba, Brazil) and water until seventeen weeks of age. Eleven male rats were randomly distributed into two groups, six control (Wistar) and five experimental (GK). The maximum caging density was four animals of the same group. A pilot study that allowed us to standardize all the procedures was conducted to minimize the number of animals used and their suffering [10,11,13]. The Animal Ethical Committee at the Cruzeiro do Sul University approved the experimental procedures (protocol number 024/2017) (Figure 1).

### 2.2. Characterization of Non-Obese T2DM Model

Five days before euthanizing the animals, the diabetes was determined by high fasting blood glucose. The rats were fasted for 12 h, a drop of blood obtained via a tail tip cut was collected onto a test strip, and their blood glucose concentrations were measured with a blood glucose monitor (AccuCheck, Corydon, IN, USA). The body weight was also measured [13].

The 120-day-old GK rats presented increased fasting blood glucose levels compared to the Wistar control group (159.3 ± 5 vs. 78.2 ± 5.5). Insulin resistance and glucose intolerance were reported in previous studies of our group [9,10,11,13], confirming the diabetic state of our colony. The final weight showed that the diabetic group had a smaller body mass (347.1 ± 3.7 vs. 508.4 ± 11) than the control.

### 2.3. Collection of Intestinal Segments

After reaching the age of 120 days, the animals were euthanized using a CO_2_ chamber and decapitated. We performed a laparotomy to collect the small and large intestine segments. The duodenum, jejunum, and distal ileum were removed, respecting the anatomical limits, as described by Pereira et al. [13]. The proximal and distal colon were removed as described by McGarrity et al. [31].

Each segment was washed with 0.1 M phosphate-buffered saline (PBS) using a needleless syringe until all its luminal content was removed and one of the ends was tied closed. The fixation process was performed using a solution of 4% PFA injected into the intestinal lumen through the free end; the volume injected promoted a slight distension of the intestine wall like that produced by an alimentary bolus [13]. The intestine-free end was tied, and the viscera was immersed in the 4% PFA solution for 5 h. After fixation, the intestinal segments were washed with saline solution and then stored at 5 °C in a refrigerator and in 15 mL conical tubes with a store solution (0.1 M PBS pH 7.4 with 0.08% sodium azide) until further use.

### 2.4. Immunofluorescence 

Whole preparations of the small intestine and colon segments were obtained by dissection under a stereomicroscope with transillumination (Bioptika, Colombo, Paraná, Brazil). The myenteric plexus was obtained by removing the submucosal tissue and mucous layer, as described by Trevizan et al. [32]. The wholemount preparations were washed twice with 0.1 M PBS solution containing 0.5% Triton X-100 for 10 min and blocked against nonspecific protein (PBS + Triton 0.5% + BSA 3%). The samples were then incubated with primary antibodies (see Table 1) for 72 h. Afterward, the intestinal segments were washed three times for 10 min, incubated with secondary antibodies (see Table 1) for 2 h, and then washed and mounted between slide and coverslip with ProLong™ Glass Antifade Mountant ^®^ mounting solution (ThermoFisher, Waltham, MA, USA, CAT#: P36980).

### 2.5. Quantitative Analysis of Myenteric Plexus

To quantify the number of neurons (HuC/D, ChAT, and nNOS) and glia (S100) per ganglion, 20 ganglia per segment and the staining of each animal were randomly photographed using a 20× objective. All neurons and glia present in the ganglia were counted. We also analyzed the percentage of ChAT+ or nNOS+ neurons normalized to the total number of Hu-IR neurons per ganglion [33]. The neuronal subpopulation and EGCs were double labeled with HuC/D for identification of myenteric ganglia, which were defined under the microscope as entities containing HuC/D+ cells separated by a distinguishable gap, as described by Lefèvre [33]. The images were obtained using a Nikon DS-Ri2 camera coupled to a Nikon Eclipse Ni-U microscope (Nikon, Tokyo, Japan), and neurons and glia per ganglion were counted using the ImageJ 1.53n software (National Institutes of Health, Bethesda, MD, USA). 

### 2.6. Morphometric Analysis of the Myenteric Plexus

For the morphometric analysis, 60 images per segment and the type of staining of each animal were randomly photographed using a 40× objective. Each image was projected onto the monitor to directly measure the neurons in a test system with inclusion and exclusion lines (according to Gundersen et al. [34]). The cell body area (μm^2^) of 100 neurons was measured per segment per staining per animal by outlining the cell body perimeter using a semi-automatic morphometric device AxioVision 4.8 (Zeiss, Jena, Germany) [13,35].

### 2.7. Statistical Analysis

All data was first tested for normal distribution with the Shapiro–Wilk test. Data with normal distribution was analyzed with the unpaired Student’s *t*-test, and the results are expressed as the mean ± SEM. The equivalent nonparametric Mann–Whitney U test was used for data with non-normal distribution, and the results are expressed as median percentiles (25%; 75%). Results with *p* values less than or equal to 0.05 were considered statistically significant. Prism 8.4.3 (GraphPad, Boston, MA, USA) was used to run the above analysis.

## 3. Results

### 3.1. Neuronal and Glial Ganglia Density 

The density of the total neuronal population per ganglion of the intestine myenteric plexus was assessed using HuC/D as a marker (Table 2). The diabetes did not provoke neuronal loss in the small intestine, a result consistent with our previous study [13]. The large intestine’s total neuronal population was also unaffected by T2DM.

**Figure 2:**
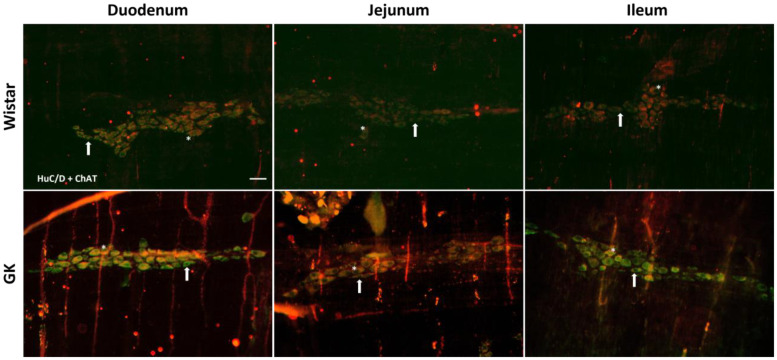
Representative photomicrograph of the results obtained by the immunofluorescence technique for the double staining HuC/D (green) + ChAT (red) of the Wistar and Goto-Kakizaki (GK) rats of 120 days of the duodenum, jejunum, and ileum. Arrows indicate HuC/D+ neurons, and asterisks indicate HuC/D + ChAT neurons. Objective: 20×. Bar: 50 μm.

**Figure 3:**
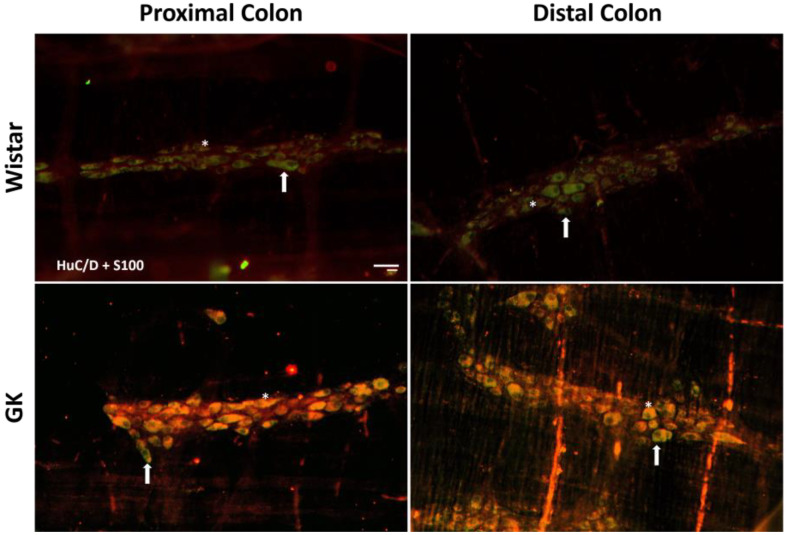
Representative photomicrograph of the results obtained by the immunofluorescence technique for the double staining HuC/D (green) + ChAT (red) of the Wistar and GK rats of 120 days of the proximal and distal colon. Arrows indicate HuC/D+ neurons, and asterisks indicate HuC/D + ChAT neurons. Objective: 20×. Bar: 50 μm.

**Figure 4:**
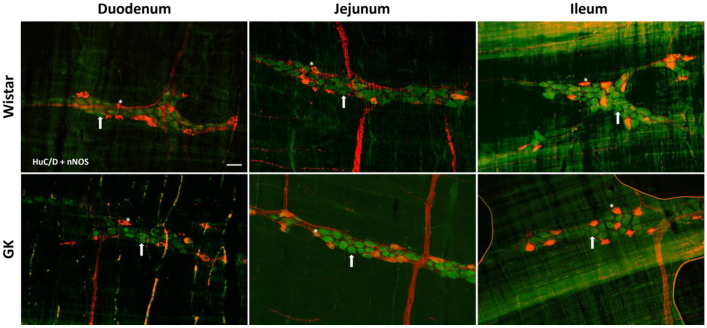
Representative photomicrograph of the results obtained by the immunofluorescence technique for the double staining HuC/D (green) + nNOS (red) of the Wistar and Goto-Kakizaki (GK) rats of 120 days of the duodenum, jejunum, and ileum. Arrows indicate HuC/D+ neurons, and asterisks indicate HuC/D + nNOS neurons. Objective: 20×. Bar: 50 μm.

**Figure 5:**
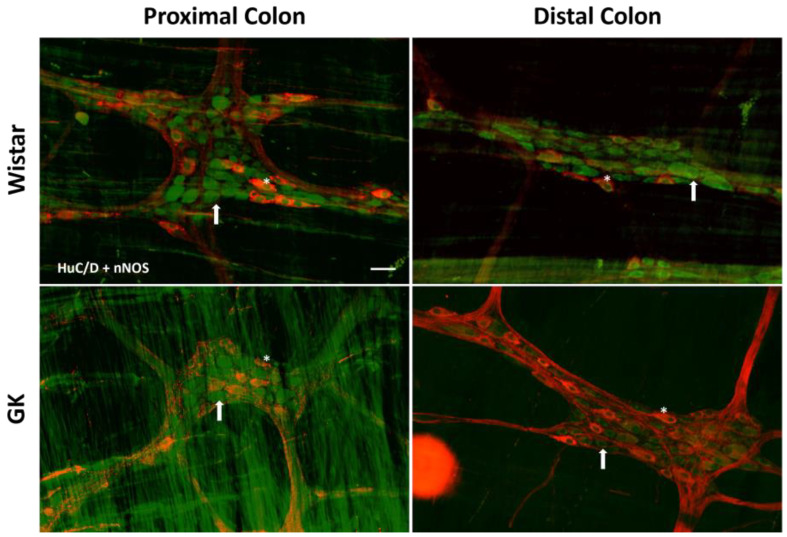
Representative photomicrograph of the results obtained by the immunofluorescence technique for the double staining HuC/D (green) + nNOS (red) of the Wistar and Goto-Kakizaki (GK) animals of 120 days of the proximal and distal colon. Arrows indicate HuC/D+ neurons, and asterisks indicate HuC/D + nNOS neurons. Objective: 20×. Bar: 50 μm.

**Figure 6:**
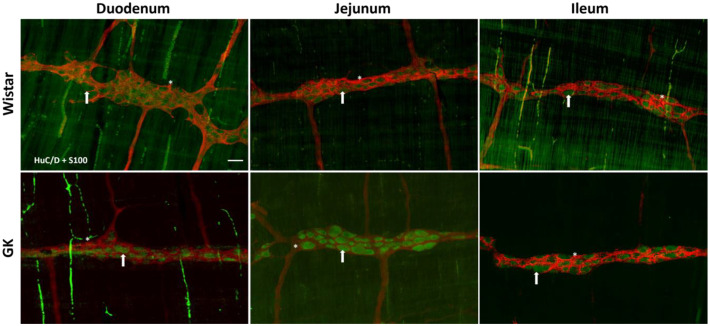
Representative photomicrograph of the results obtained by the immunofluorescence technique for the double staining HuC/D (green) + S100 (red) of the Wistar and Goto-Kakizaki (GK) rats of 120 days of the duodenum, jejunum, and ileum. Arrows indicate HuC/D+ neurons, and asterisks indicate enteric glial cells. Objective: 20×. Bar: 50 μm.

**Figure 7:**
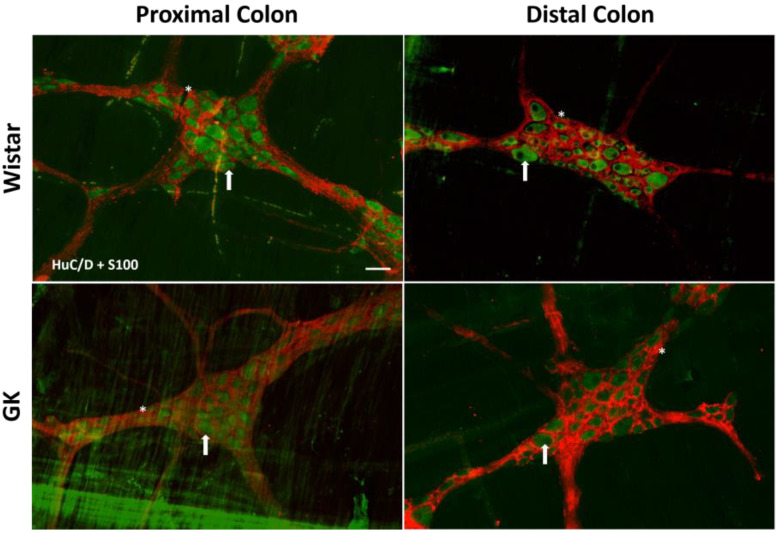
Representative photomicrograph of the results obtained by the immunofluorescence technique for the double staining HuC/D (green) + S100 (red) of the Wistar and Goto-Kakizaki (GK) rats of 120 days of the proximal and distal colon. Arrows indicate HuC/D+ neurons, and asterisks indicate enteric glial cells. Objective: 20×. Bar: 50 μm.

There was a significant reduction in the excitatory (ChAT+) neuronal density in the small intestine of the GK rats: 38.8% in the duodenum (*p* < 0.001), 20.4% in the jejunum (*p* < 0.01), and 22.3% in the ileum (*p* < 0.01) compared to the corresponding intestinal segments from the Wistar control rats (Figure 2). In contrast, ChAT+ density was 20% higher (*p* < 0.05) in the proximal colon of the GK rats compared to the control animals (Figure 3). The nitrergic neuron density in the small and large intestines of both the GK and Wistar control rats was similar (Figure 4 and Figure 5). Additionally, the EGC density (i.e., the number of glial cells per ganglia) in the ileum was increased by 38.7% in the GK rats compared to the controls (*p* < 0.01) (Figure 6). Still, no differences were detected in the large intestine (Figure 7).

The neuronal density was also evaluated as a percentage of ChAT+ or nNOS+ neurons normalized to the total number of Hu-IR neurons per ganglion. Concerning the percentages of excitatory neurons in the small intestine, we observed a decrease in the diabetic GK rats in the duodenum (60.7% vs. 87.4%), jejunum (55.3% vs. 72.8%), and ileum (58.3% vs. 72%). In the large intestine of the GK rats, the percentages of cholinergic neurons were increased in the proximal colon compared to the control animals (78.5% vs. 68.5%). In contrast, in the distal colon, the percentages were 66.7% and 72.8% in the GK and Wistar control rats, respectively.

The percentages of nitrergic neurons normalized to the total neurons per ganglion in the GK and Wistar control rats were similar in the duodenum (19.7% vs. 16.8%), jejunum (23.7% vs. 20.3%), and ileum (21.5% vs. 21.8%). There were also no alterations in the percentages of nNOS+ neurons in the proximal colon of the GK and Wistar control rats (18.7% vs. 20.5%) or in the distal colon (22.2% vs. 22.6%).

### 3.2. Morphometric Analysis of Neuronal Cell Body Area 

The morphometric analysis of the cell body area of myenteric neurons (Table 3) confirms our previous study [13]. The body area of HuC/D+ neurons was increased in the jejunum (29%, *p* < 0.05) and ileum (26%, *p* < 0.01) of the GK rats, and no differences were observed in the proximal and distal colons. We then investigated the contribution of neuronal subpopulation in the neuronal hypertrophy reported in the total population. Cell body areas of cholinergic and nitrergic neurons were not statistically different between small and large intestine groups.

**Figure 8:**
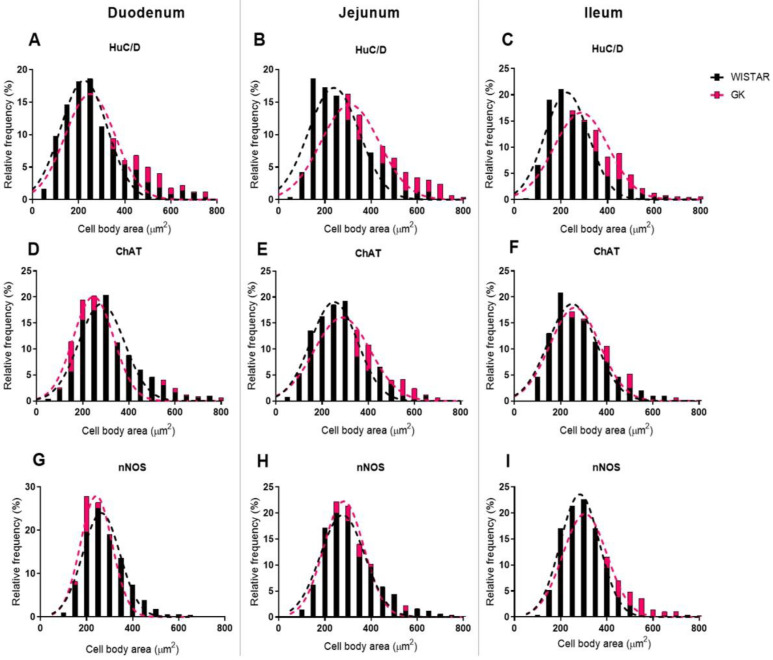
Frequency histogram (%) of the cell body area (µm^2^) of the myenteric neurons of the small intestine of 120-day-old animals of the HuC/D (**A**–**C**), ChAT (**D**–**F**) and nNOS populations (**G**–**I**). The number of animals used was 4 to 5.

However, the analysis of cell body area in frequency histograms suggested that the larger cell body area in the jejunum is due to the contribution of cholinergic neurons, of which 30% had an area between 350 µm^2^ and 450 µm^2^ (Figure 8E). In contrast, in the control group, 20.7% of the neurons were in this area range. The increased area seen in the ileum of the GK rats is likely due to an increased occurrence of nitrergic neurons with areas above 450 µm^2^ (Figure 8I).

**Figure 9:**
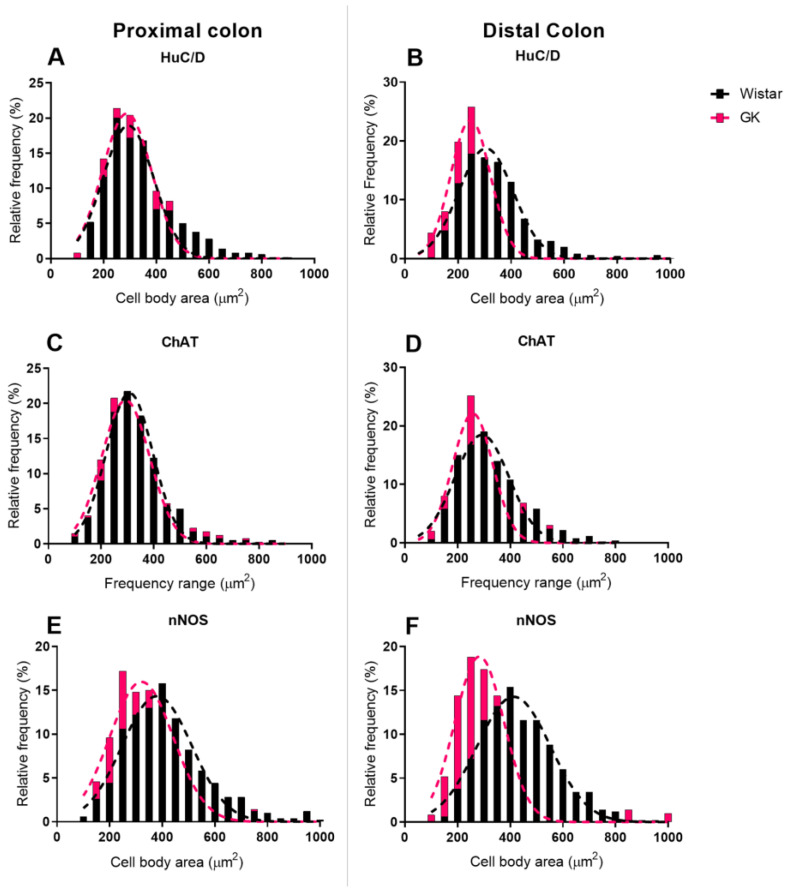
Frequency histogram (%) of the cell body area (µm^2^) of the myenteric neurons of the large intestine of 120-day-old animals of the HuC/D (**A**,**B**), ChAT (**C**,**D**) and nNOS populations (**E**,**F**). The number of animals used was 4 to 5.

The myenteric neuron body areas (µm^2^) were depicted in histograms (Figure 8 and Figure 9) and presented as relative frequency (percentage). The areas of the three segments of the small intestine of the Wistar group varied between 150 and 250 µm^2^, whereas, in the GK animals, it ranged from 200 to 250 µm^2^ in the duodenum and 200 to 350 µm^2^ in the jejunum and ileum for the general population. In the proximal colon of both groups and the distal colon of the Wistar rats, the areas ranged from 250 to 350 µm^2^. Moreover, the GK rats exhibited cell body areas between 200 and 300 µm^2^ in the distal colon (Figure 9).

ChAT+ neuron areas ranged from 200 to 300 µm^2^ in the duodenum and ileum of both groups. In the jejunum, the GK rats exhibited areas within the range of 250 to 300 µm^2^, while for the Wistar rats, the areas were from 200 to 300 µm^2^. In the proximal colon, ChAT+ neurons had a similar area distribution in both groups, from 250 to 350 µm^2^. In the distal colon, areas between 200 and 300 µm^2^ prevailed in the GK and control groups.

Regarding the nitrergic subpopulation, the neurons displayed a similar distribution in the duodenum and jejunum, with areas between 200 and 300 µm^2^. In the ileum, the myenteric neurons of the Wistar rats had areas between 200 and 350 µm^2^, whereas in the GK group, around 55% of the neurons had areas between 250 and 350 µm^2^. In the ileum, some nitrergic neuron areas of the GK rats were greater than 600 µm^2^, which was not observed in controls. The Wistar group had areas between 300 and 400 µm^2^ in the proximal colon. In the GK rats, neurons had areas between 250 and 350 µm^2^ in the proximal colon and 200 and 350 µm^2^ in the distal colon. In the distal colon of the control group, areas between 300 and 500 µm^2^ prevailed.

## 4. Discussion

The present study is the first to characterize the myenteric neuron subpopulations and evaluate the EGCs in GK rats’ small and large intestines. Although no differences were detected in the neuronal density of the general population or the nitrergic neurons, the cholinergic subpopulation exhibited fewer neurons per ganglion in the three segments of the small intestine and an increased number in the proximal colon of the GK rats compared to the Wistar control group. The number of EGCs was increased in the ileum of the GK rats. The area of the cell body was increased in the total neuronal population of the jejunum and ileum of the GK group. The distribution of cell body areas in frequency histograms pointed out a contribution of cholinergic neurons to larger regions in the jejunum and nitrergic neurons in the ileum.

The loss of myenteric neurons has been reported in the small and large intestines of diet- and streptozotocin-induced DM models [36,37,38]. Herein, we reported preservation of HuC/D+ in the small intestine, as previously described by our group, and in the large intestine, with no difference in the number of neurons per ganglia when compared with the control group. Diabetes-induced damage to enteric neurons is associated with the duration and glycemic control [18] and can damage the neuronal subpopulations differently.

The nNOS+ subpopulation is the most susceptible to neuronal density changes in diabetic models [39]. Decreases in nitrergic neurons have been detected in the duodenum of high-fat diet (HFD) mice, the jejunum of biobreeding diabetic rats, and the distal ileum of rats with T1DM [29,40,41]. In the present study, we did not observe changes in the nitrergic inhibitory neuron density in the small and large intestines of the GK rats, only alterations within the cell body area. However, the reported DM-associated ENS impairment in streptozotocin- and diet-induced models may be due to drug toxicity or the obesity background rather than diabetes per se.

The density of the excitatory cholinergic neuron subpopulation was decreased in the entire small intestine and increased in the proximal colon of the GK rats. Reduced cholinergic neuron density in the distal ileum was reported in female rats with streptozotocin-induced DM [29]. Notably, attenuated cholinergic function (i.e., impairment of ChAT and acetylcholine release) was associated with constipation by impairing the electrical circuit involved in motility [42,43,44,45].

Studies have pointed out the participation of cholinergic signaling in regulating inflammation and preserving motility since the cholinergic anti-inflammatory pathway is known to suppress the release of cytokine [46,47]. Therefore, the increase in the cholinergic neurons in the proximal colon of GK rats could be not only a compensatory mechanism to counterbalance the decrease of ChAT+ neurons in the small intestine but also a neuroplastic response to gut inflammation. Enteric neuroplasticity is an adaptative response in the morphology or chemical code of enteric neurons and glia to maintain gut function [25,48,49].

We also observed morphological changes in the myenteric neurons. The general population of jejunum and ileum exhibited increased neuron body areas, with increases of 29% and 26%, respectively. This result was also previously reported by our group [13]. While the cell body area of cholinergic and nitrergic neurons was not statistically different from the control group in the small and large intestines, the analysis of frequency histograms suggested that the larger cell body area was due to increased contributions from the cholinergic neurons in the jejunum. The augmented nitrergic subpopulation in the ileum appears to support the increased area values.

The increase in cell body area in the diabetic state is often associated with oxidative stress since administering substances with antioxidant properties prevents neuronal hypertrophy [50,51]. For example, administering microcapsules containing quercetin to streptozotocin-induced diabetic Wistars rats was associated with a neuroprotective effect, preserving nitrergic neurons and reducing neuron body area [52]. Other studies reported similar results with antioxidant compounds [50,51].

In the ileum of the GK rats, there was an increased number of glial cells per ganglia. The increase in glial density was reported in diet-induced DM in the duodenum [28] and the colon [53]. The enteric glial is crucial for handling and supporting the enteric neurons and maintaining tissue homeostasis [54]. Glia cells communicate with immune cells and play a role in neuroinflammation and motility control. 

The increase in S100β expression is associated with enteric gliosis and the release of glial mediators, such as cytokines [53,55,56]. We previously reported increased inflammatory markers such as IL-1β and NF-κB in the small intestine of GK rats [13]. As glial cells secrete IL-1β [57,58], the increased glial density could react to the inflammation state induced by DM within the ENS. The S100β protein can also bind to the advanced glycation end products receptor (RAGE) [59]. Upregulated RAGE expression in GK rats’ intestines was reported [60]. Although the area of enteric glia was not measured in the present study, the increase in the S100+ cell density indicates enteric gliosis [61] in the ileum of GK rats. These latter findings support the involvement of reactive glia in response to metabolic and inflammatory changes in the enteric microenvironment provoked by T2DM.

The participation of the gut has been highlighted in many diseases, including T2DM [62]. The enteric neurons are glucoresponsive [63] and participate in enteroendocrine and enterocyte communication and the intestinal transport of glucose [64]. The ENS is the master controller of gut functions, and evidence strongly suggests that enteric neuron and glial cell impairment is linked to DM effects and gastrointestinal symptoms in diabetic patients [39,62,65] and in IR [30]. Even though constipation is one of the most prevalent symptoms reported in diabetic patients, this condition is often overlooked [66].

## 5. Conclusions

In the present study, we used the GK rat to investigate the DM-induced effects without the interferences traditional DM models require, such as drug administration and obesogenic diets. This study is the first to characterize the main neuronal subpopulation and EGCs of the myenteric plexus from the small and large intestines of these diabetic animals. Notably, we identified specific alterations in the density of myenteric cholinergic neurons with possible neuroplastic response and enteric gliosis that could contribute to the delayed GI transit and intestinal inflammation previously reported by our group in this non-obese T2DM model.

## Figures and Tables

**Table 1:** List of primary and secondary antibodies for immunofluorescence assays.

Antibodies	Dilution	Source	Identifier
Mouse anti-HuC/D	1:500	Invitrogen, Carlsbad, CA, USA	CAT#: A21271
Rabbit anti-nNOS	1:500	Abcam, Cambridge, UK	CAT#: ab76067
Goat anti-ChAT	1:40	Millipore, MA, USA	CAT#: AB144P
Rabbit anti-S100 beta	1:1000	Abcam, Cambridge, UK	CAT#: ab52642
Alexa Fluor 488 donkey anti-mouse IgG	1:500	Invitrogen, Carlsbad, CA, USA	CAT#: A21202
Alexa Fluor 596 goat anti-rabbit IgG	1:500	Abcam, Cambridge, UK	CAT#: ab150080
Alexa Fluor 546 donkey anti-goat IgG	1:500	Invitrogen, Carlsbad, CA, USA	CAT#: A11056

**Table 2:** Analysis of neuron and glial density per ganglion of the myenteric plexus of the small and large intestines of 120-day-old Wistar control and GK rats.

	Duodenum		Jejunum		Ileum		Proximal Colon		Distal Colon	
	Wistar	GK	Wistar	GK	Wistar	GK	Wistar	GK	Wistar	GK
HuC/D	36 ± 2	32 ± 1.4	32 (31; 36.5)	33 (30.5; 40)	37 ± 1.1	36 ± 1	41.8 ± 1.2	43.8 ± 2.3	42.6 ± 3.4	46.4 ± 2.7
S100	16.3 ± 2.5	14.7 ± 1.4	13.4 ± 1.1	13.5 ± 0.7	12.9 ± 0.5	17.9 ± 1 **	25.2 ± 1.3	22.4 ± 1.2	20.5 ± 1.4	22.3 ± 2
ChAT	31.7 ± 2	19.4 ± 0.8 ***	24.4 ± 1.2	19.4 ± 0.75 **	26.9 ± 1.1	20.9 ± 0.7 **	28.6 ± 1.3	34.4 ± 1.3 *	31 ± 2.8	30.9 ± 2.1
nNOS	6.1 ± 0.45	6.3 ± 0.26	6.8 ± 0.44	8.2 ± 0.59	8.1 ± 0.7	7.7 ± 0.8	8.5 ± 0.6	8.2 ± 0.9	9.6 ± 1.3	10.3 ± 0.9

Data compared using the Student’s *t*-test are expressed as the mean ± SEM. Data compared using the Mann–Whitney test are expressed as median percentiles (25%; 75%). (*) *p* < 0.05; (**) *p* < 0.01 (***) *p* < 0.001. The number of animals used was 4 to 6.

**Table 3:** Analysis of the cell body area (µm^2^) from the myenteric plexus neurons in the small and large intestine of 120-day-old Wistar control and GK rats.

	Duodenum		Jejunum		Ileum		Proximal Colon		Distal Colon	
	Wistar	GK	Wistar	GK	Wistar	GK	Wistar	GK	Wistar	GK
HuC/D	268 ± 35.6	315.6 ± 15.5	282 ± 28.3	366 ± 10.5 *	255.7 ± 22.8	323.4 ± 14.8 **	339.5 ± 12.8	318 ± 10.3	336 ± 19.9	284 ± 11.6
ChAT	322 ± 21	300 ± 12.8	277 ± 21.5	318 ± 12.2	280 ± 15.9	296 ± 12.5	329 ± 25.3	325 ± 13.9	327 ± 20.8	306 ± 13.9
nNOS	285 ± 9.3	262 ± 14	312 ± 20.2	302 ± 21.6	295 ± 8.3	338 ± 27.4	420 ± 24.5	361.5± 29.3	437 (403.5; 468.5)	290 (272; 460.6)

Data compared using the Student’s *t*-test are expressed as the mean ± SEM. Data compared using the Mann–Whitney test are expressed as median percentiles (25%; 75%). (*) *p* < 0.05; (**) *p* < 0.01. The number of animals used was 4 to 5.

## Data Availability

The authors will make the raw data supporting this article’s conclusions available upon request.

## References

[1] American Diabetes Association Professional Practice Committee (2022). 2. Classification and Diagnosis of Diabetes: Standards of Medical Care in Diabetes—2022. Diabetes Care.

[2] Aroda V.R., Krause-Steinrauf H., Kazemi E.J., Buse J.B., Gulanski B.I., Florez H.J., Ahmann A.J., Loveland A., Kuhn A., Lonier J.Y. (2022). Clinical and Metabolic Characterization of Adults With Type 2 Diabetes by Age in the Glycemia Reduction Approaches in Diabetes: A Comparative Effectiveness Study (GRADE) Cohort. Diabetes Care.

[3] International Diabetes Federation (2021). IDF Diabetes Atlas.

[4] Acosta-Martinez M., Cabail M.Z. (2022). The PI3K/Akt Pathway in Meta-Inflammation. Int. J. Mol. Sci..

[5] Johnson A.R., Justin Milner J., Makowski L. (2012). The Inflammation Highway: Metabolism Accelerates Inflammatory Traffic in Obesity. Immunol. Rev..

[6] Russo S., Kwiatkowski M., Govorukhina N., Bischoff R., Melgert B.N. (2021). Meta-Inflammation and Metabolic Reprogramming of Macrophages in Diabetes and Obesity: The Importance of Metabolites. Front. Immunol..

[7] Goto Y., Kakizaki M., Masaki N. (1975). Spontaneous Diabetes Produced by Selective Breeding of Normal Wistar Rats. Proc. Jpn. Acad..

[8] Goto Y., Kakizaki M., Masaki N. (1976). Production of Spontaneous Diabetic Rats by Repetition of Selective Breeding. Tohoku J. Exp. Med..

[9] Kuwabara W.M.T., Panveloski-Costa A.C., Yokota C.N.F., Pereira J.N.B., Filho J.M., Torres R.P., Hirabara S.M., Curi R., Alba-Loureiro T.C. (2017). Comparison of Goto-Kakizaki Rats and High Fat Diet-Induced Obese Rats: Are They Reliable Models to Study Type 2 Diabetes Mellitus?. PLoS ONE.

[10] Serdan T.D.A., Masi L.N., Pereira J.N.B., Rodrigues L.E., Alecrim A.L., Scervino M.V.M., Diniz V.L.S., dos Santos A.A.C., Filho C.P.B.S., Alba-Loureiro T.C. (2021). Impaired Brown Adipose Tissue Is Differentially Modulated in Insulin-Resistant Obese Wistar and Type 2 Diabetic Goto-Kakizaki Rats. Biomed. Pharmacother..

[11] Borges J.C.O., Oliveira V.A.B., Serdan T.D.A., Silva F.L.R., Santos C.S., Pauferro J.R.B., Ribas A.S.F., Manoel R., Pereira A.C.G., Correa I.S. (2023). Brain Glucose Hypometabolism and Hippocampal Inflammation in Goto-Kakizaki Rats. Braz. J. Med. Biol. Res..

[12] Gimenes G.M., Santana G.O., Scervino M.V.M., Curi R., Pereira J.N.B. (2022). A Short Review on the Features of the Non-Obese Diabetic Goto-Kakizaki Rat Intestine. Braz. J. Med. Biol. Res..

[13] Pereira J.N.B., Murata G.M., Sato F.T., Marosti A.R., Carvalho C.R.d.O., Curi R. (2021). Small Intestine Remodeling in Male Goto–Kakizaki Rats. Physiol. Rep..

[14] Ouyang X., Li S., Tan Y., Lin L., Yin J., Chen J.D.Z. (2019). Intestinal Electrical Stimulation Attenuates Hyperglycemia and Prevents Loss of Pancreatic β Cells in Type 2 Diabetic Goto–Kakizaki Rats. Nutr. Diabetes.

[15] Chandrasekharan B., Anitha M., Blatt R., Shahnavaz N., Staley C., Mwangi S., Jones D.P., Sitaraman S.V. (2012). Colonic Motor Dysfunction in Human Diabetes Is Associated with Enteric Neuronal Loss and Increased Oxidative Stress. Neurogastroenterol. Motil..

[16] Maisey A. (2016). A Practical Approach to Gastrointestinal Complications of Diabetes. Diabetes Ther..

[17] Krishnan B., Babu S., Walker J., Walker A.B., Pappachan J.M. (2013). Gastrointestinal Complications of Diabetes Mellitus. World J. Diabetes.

[18] Meldgaard T., Brock C. (2019). Diabetes and the Gastrointestinal Tract. Medicine.

[19] Furness J.B., Stebbing M.J. (2018). The First Brain: Species Comparisons and Evolutionary Implications for the Enteric and Central Nervous Systems. Neurogastroenterol. Motil..

[20] Furness J.B. (2000). Types of Neurons in the Enteric Nervous System. J. Auton. Nerv. Syst..

[21] Furness J.B. (2006). The Organisation of the Autonomic Nervous System: Peripheral Connections. Auton. Neurosci..

[22] Furness J.B. (2012). The Enteric Nervous System and Neurogastroenterology. Nat. Rev. Gastroenterol. Hepatol..

[23] Olsson C., Holmgren S. (2001). The Control of Gut Motility. Comp. Biochem. Physiol. A Mol. Integr. Physiol..

[24] Abot A., Lucas A., Bautzova T., Bessac A., Fournel A., Le-Gonidec S., Valet P., Moro C., Cani P.D., Knauf C. (2018). Galanin Enhances Systemic Glucose Metabolism through Enteric Nitric Oxide Synthase-Expressed Neurons. Mol. Metab..

[25] Sharkey K.A., Mawe G.M. (2023). The Enteric Nervous System. Physiol. Rev..

[26] Coelho-Aguiar J.d.M., Bon-Frauches A.C., Gomes A.L.T., Veríssimo C.P., Aguiar D.P., Matias D., Thomasi B.B.d.M., Gomes A.S., Brito G.A.d.C., Moura-Neto V. (2015). The Enteric Glia: Identity and Functions. Glia.

[27] Bessac A., Cani P.D., Meunier E., Dietrich G., Knauf C. (2018). Inflammation and Gut-Brain Axis during Type 2 Diabetes: Focus on the Crosstalk between Intestinal Immune Cells and Enteric Nervous System. Front. Neurosci..

[28] McMenamin C.A., Clyburn C., Browning K.N. (2018). High-Fat Diet During the Perinatal Period Induces Loss of Myenteric Nitrergic Neurons and Increases Enteric Glial Density, Prior to the Development of Obesity. Neuroscience.

[29] Brasileiro A.D., Garcia L.P., de Carvalho da Silva S., Rocha L.B., Pedrosa A.L., Vieira A.S., da Silva V.J.D., Rodrigues A.R.A. (2019). Effects of Diabetes Mellitus on Myenteric Neuronal Density and Sodium Channel Expression in the Rat Ileum. Brain Res..

[30] Fournel A., Drougard A., Duparc T., Marlin A., Brierley S.M., Castro J., Le-Gonidec S., Masri B., Colom A., Lucas A. (2017). Apelin Targets Gut Contraction to Control Glucose Metabolism via the Brain. Gut.

[31] McGarrity T.J., Peiffer L.P., Colony P.C. (1988). Cellular Proliferation in Proximal and Distal Rat Colon during 1,2-Dimethylhydrazine-Induced Carcinogenesis. Gastroenterology.

[32] Trevizan A.R., Schneider L.C.L., Araújo E.J.d.A., Garcia J.L., Buttow N.C., Nogueira-Melo G.d.A., Sant’Ana D.d.M.G. (2019). Acute Toxoplasma Gondii Infection Alters the Number of Neurons and the Proportion of Enteric Glial Cells in the Duodenum in Wistar Rats. Neurogastroenterol. Motil..

[33] Lefèvre C., Bessard A., Aubert P., Joussain C., Giuliano F., Behr-Roussel D., Perrouin-Verbe M.-A., Perrouin-Verbe B., Brochard C., Neunlist M. (2020). Enteric Nervous System Remodeling in a Rat Model of Spinal Cord Injury: A Pilot Study. Neurotrauma Rep..

[34] Gundersen H.J.G., Bendtsen T.F., Korbo L., Marcussen N., Møller A., Nielsen K., Nyengaard J.R., Pakkenberg B., Sørensen F.B., Vesterby A. (1988). Some New, Simple and Efficient Stereological Methods and Their Use in Pathological Research and Diagnosis. APMIS.

[35] Pereira J.N.B., Mari R.B., Stabille S.R., De Faria H.G., Mota T.F.M., Ferreira W.M. (2014). Benefits of Caloric Restriction in the Myenteric Neuronal Plasticity in Aging Rats. An. Acad. Bras. Cienc..

[36] Stenkamp-Strahm C.M., Kappmeyer A.J., Schmalz J.T., Gericke M., Balemba O. (2013). High-Fat Diet Ingestion Correlates with Neuropathy in the Duodenum Myenteric Plexus of Obese Mice with Symptoms of Type 2 Diabetes. Cell Tissue Res..

[37] Beraldi E.J., Soares A., Borges S.C., de Souza A.C.d.S., Natali M.R.M., Bazotte R.B., Buttow N.C. (2015). High-Fat Diet Promotes Neuronal Loss in the Myenteric Plexus of the Large Intestine in Mice. Dig. Dis. Sci..

[38] Izbéki F., Wittman T., Rosztóczy A., Linke N., Bódi N., Fekete É., Bagyánszki M. (2008). Immediate Insulin Treatment Prevents Gut Motility Alterations and Loss of Nitrergic Neurons in the Ileum and Colon of Rats with Streptozotocin-Induced Diabetes. Diabetes Res. Clin. Pract..

[39] Bagyánszki M. (2012). Diabetes-Related Alterations in the Enteric Nervous System and Its Microenvironment. World J. Diabetes.

[40] Nyavor Y., Brands C.R., May G., Kuther S., Nicholson J., Tiger K., Tesnohlidek A., Yasuda A., Starks K., Litvinenko D. (2020). High-Fat Diet–Induced Alterations to Gut Microbiota and Gut-Derived Lipoteichoic Acid Contributes to the Development of Enteric Neuropathy. Neurogastroenterol. Motil..

[41] Demedts I., Masaoka T., Kindt S., De Hertogh G., Geboes K., Farré R., Berghe P.V., Tack J. (2013). Gastrointestinal Motility Changes and Myenteric Plexus Alterations in Spontaneously Diabetic Biobreeding Rats. J. Neurogastroenterol. Motil..

[42] Gros M., Gros B., Mesonero J.E., Latorre E. (2021). Neurotransmitter Dysfunction in Irritable Bowel Syndrome: Emerging Approaches for Management. J. Clin. Med..

[43] Deb B., Prichard D.O., Bharucha A.E. (2020). Constipation and Fecal Incontinence in the Elderly. Curr. Gastroenterol. Rep..

[44] Fornai M., Pellegrini C., Antonioli L., Segnani C., Ippolito C., Barocelli E., Ballabeni V., Vegezzi G., Al Harraq Z., Blandini F. (2016). Enteric Dysfunctions in Experimental Parkinsons Disease: Alterations of Excitatory Cholinergic Neurotransmission Regulating Colonic Motility in Rats. J. Pharmacol. Exp. Ther..

[45] Xu J., Wang L., Chen X., Le W. (2022). New Understanding on the Pathophysiology and Treatment of Constipation in Parkinson’s Disease. Front. Aging Neurosci..

[46] Rahman A.A., Stavely R., Pan W., Ott L., Ohishi K., Ohkura T., Han C., Hotta R., Goldstein A.M. (2024). Optogenetic Activation of Cholinergic Enteric Neurons Reduces Inflammation in Experimental Colitis. CMGH.

[47] Spear E.T., Mawe G.M. (2019). MINI-REVIEW Neurogastroenterology and Motility Enteric Neuroplasticity and Dysmotility in Inflammatory Disease: Key Players and Possible Therapeutic Targets. Am. J. Physiol. Gastrointest. Liver Physiol..

[48] Lomax A.E., Fernández E., Sharkey K.A. (2005). Plasticity of the Enteric Nervous System during Intestinal Inflammation. Neurogastroenterol. Motil..

[49] Vasina V., Barbara G., Talamonti L., Stanghellini V., Corinaldesi R., Tonini M., De Ponti F., De Giorgio R. (2006). Enteric Neuroplasticity Evoked by Inflammation. Auton. Neurosci..

[50] Ferreira P.E.B., Beraldi E.J., Borges S.C., Natali M.R.M., Buttow N.C. (2018). Resveratrol Promotes Neuroprotection and Attenuates Oxidative and Nitrosative Stress in the Small Intestine in Diabetic Rats. Biomed. Pharmacother..

[51] de Souza S.R.G., Neto M.H.d.M., Perles J.V.C.M., Frez F.C.V., Zignani I., Ramalho F.V., Hermes-Uliana C., Bossolani G.D.P., Zanoni J.N. (2017). Antioxidant Effects of the Quercetin in the Jejunal Myenteric Innervation of Diabetic Rats. Front. Med..

[52] Sehaber-Sierakowski C.C., Vieira-Frez F.C., Hermes-Uliana C., Martins H.A., Bossolani G.D.P., Lima M.M., Blegniski F.P., Guarnier F.A., Baracat M.M., Perles J.V.C.M. (2021). Protective Effects of Quercetin-Loaded Microcapsules on the Enteric Nervous System of Diabetic Rats. Auton. Neurosci..

[53] Antonioli L., D’Antongiovanni V., Pellegrini C., Fornai M., Benvenuti L., di Carlo A., van den Wijngaard R., Caputi V., Cerantola S., Giron M.C. (2020). Colonic Dysmotility Associated with High-fat Diet-induced Obesity: Role of Enteric Glia. FASEB J..

[54] Ochoa-Cortes F., Turco F., Linan-Rico A., Soghomonyan S., Whitaker E., Wehner S., Cuomo R., Christofi F.L. (2016). Enteric Glial Cells: A New Frontier in Neurogastroenterology and Clinical Target for Inflammatory Bowel Diseases. Inflamm. Bowel Dis..

[55] Cirillo C., Sarnelli G., Esposito G., Turco F., Steardo L., Cuomo R. (2011). S100B Protein in the Gut: The Evidence for Enteroglialsustained Intestinal Inflammation. World J. Gastroenterol..

[56] Linan-Rico A., Ochoa-Cortes F., Schneider R., Christofi F.L. (2023). Mini-Review: Enteric Glial Cell Reactions to Inflammation and Potential Therapeutic Implications for GI Diseases, Motility Disorders, and Abdominal Pain. Neurosci. Lett..

[57] Pochard C., Coquenlorge S., Freyssinet M., Naveilhan P., Bourreille A., Neunlist M., Rolli-Derkinderen M. (2018). The Multiple Faces of Inflammatory Enteric Glial Cells: Is Crohn’s Disease a Gliopathy?. Am. J. Physiol. Gastrointest. Liver Physiol..

[58] Murakami M., Ohta T., Ito S. (2009). Lipopolysaccharides Enhance the Action of Bradykinin in Enteric Neurons via Secretion of Interleukin-1β from Enteric Glial Cells. J. Neurosci. Res..

[59] Costa D.V.S., Moura-Neto V., Bolick D.T., Guerrant R.L., Fawad J.A., Shin J.H., Medeiros P.H.Q.S., Ledwaba S.E., Kolling G.L., Martins C.S. (2021). S100B Inhibition Attenuates Intestinal Damage and Diarrhea Severity During Clostridioides Difficile Infection by Modulating Inflammatory Response. Front. Cell Infect. Microbiol..

[60] Chen P.-M., Gregersen H., Zhao J.-B. (2015). Advanced Glycation End-Product Expression Is Upregulated in the Gastrointestinal Tract of Type 2 Diabetic Rats. World J. Diabetes.

[61] López-Gómez L., Szymaszkiewicz A., Zielińska M., Abalo R. (2021). Nutraceuticals and Enteric Glial Cells. Molecules.

[62] Meldgaard T., Olesen S.S., Farmer A.D., Krogh K., Wendel A.A., Brock B., Drewes A.M., Brock C. (2018). Diabetic Enteropathy: From Molecule to Mechanism-Based Treatment. J. Diabetes Res..

[63] Liu M.-T., Seino S., Kirchgessner A.L. (1999). Identification and Characterization of Glucoresponsive Neurons in the Enteric Nervous System. J. Neurosci..

[64] Moran A.W., Al-Rammahi M.A., Batchelor D.J., Bravo D.M., Shirazi-Beechey S.P. (2018). Glucagon-Like Peptide-2 and the Enteric Nervous System Are Components of Cell-Cell Communication Pathway Regulating Intestinal Na+/Glucose Co-Transport. Front. Nutr..

[65] Meldgaard T., Keller J., Olesen A.E., Olesen S.S., Krogh K., Borre M., Farmer A., Brock B., Brock C., Drewes A.M. (2019). Pathophysiology and Management of Diabetic Gastroenteropathy. Therap. Adv. Gastroenterol..

[66] Abdu Seid M., Diress M., Mohammed A., Sinamaw D. (2023). Chronic Constipation and Its Associated Factors in Patients with Type-2 Diabetes: A Multicenter Cross-Sectional Study. Diabetes Res. Clin. Pract..

